# Supplementary material for: Selective weakening of population-coupled synaptic activity in vivo in a mouse model of amyloid-beta pathology
Source: Nat Commun. 2026 Mar 7;17:3646. doi: 10.1038/s41467-026-69866-3 (PMC13096637; doi:10.1038/s41467-026-69866-3)
Supplement: Supplementary file 1 — Supplementary Information [file 41467_2026_69866_MOESM1_ESM.pdf]

Supplementary information

Supplementary Figures

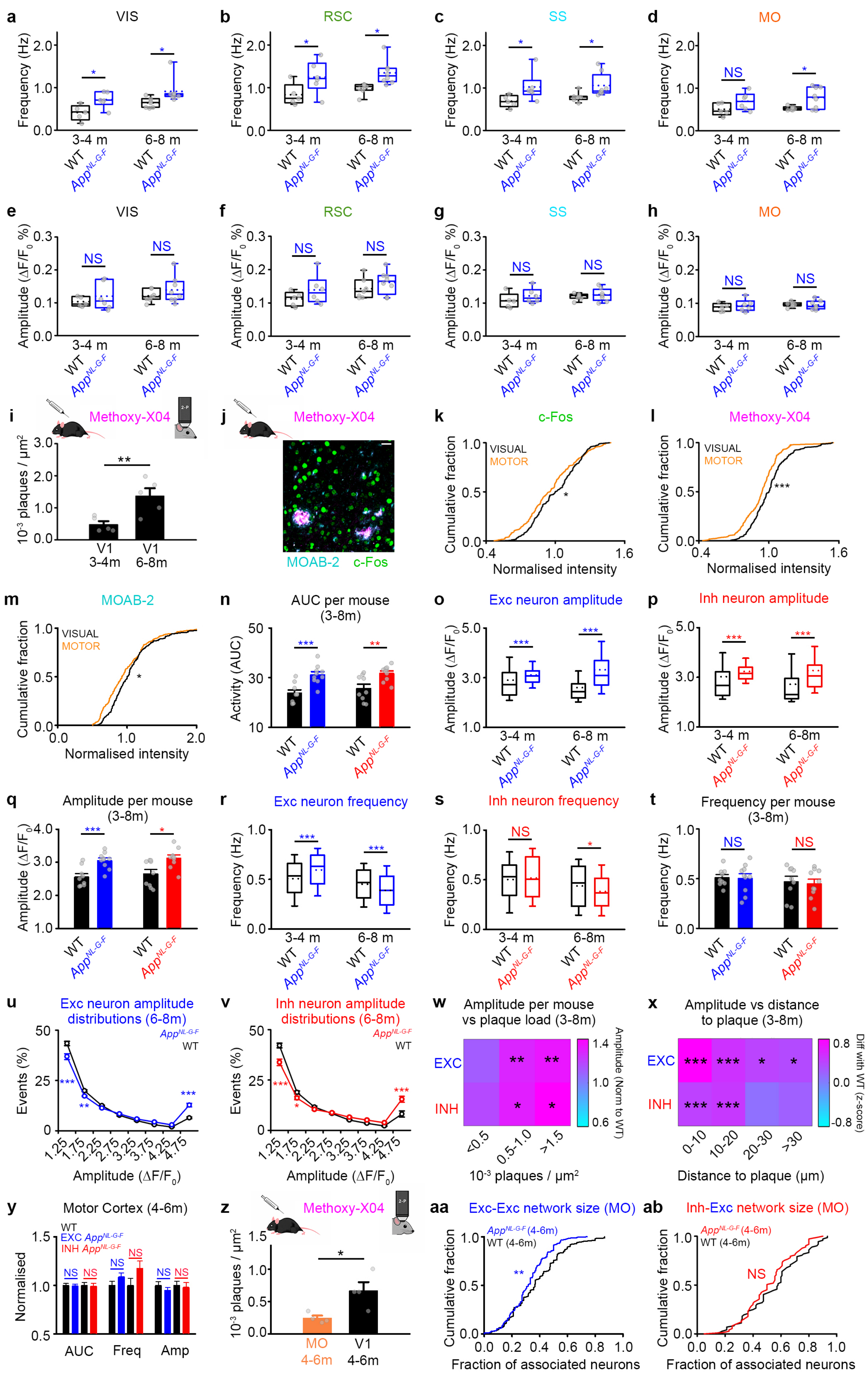

a VIS
b RSC
c SS
d MO
Frequency (Hz)
2.0
1.0
0.0
*
NS
3-4 m
6-8 m
WT
App^NL-G-F
e VIS
f RSC
g SS
h MO
Amplitude (ΔF/F0 %)
0.3
0.2
0.1
0.0
NS
i Methoxy-X04
10^-3 plaques / μm^2
3.0
2.0
1.0
0.0
**
V1 3-4m
V1 6-8m
j Methoxy-X04
MOAB-2
c-Fos
k c-Fos
VISUAL
MOTOR
Cumulative fraction
1.0
0.5
0.0
0.4
1.0
1.6
Normalised intensity
l Methoxy-X04
***
m MOAB-2
0.0
1.0
2.0
n AUC per mouse (3-8m)
Activity (AUC)
50
30
10
***
**
o Exc neuron amplitude
Amplitude (ΔF/F0)
5.0
3.0
1.0
p Inh neuron amplitude
3-4 m
6-8m
q Amplitude per mouse (3-8m)
4.0
3.0
2.0
1.0
r Exc neuron frequency
s Inh neuron frequency
t Frequency per mouse (3-8m)
0.5
u Exc neuron amplitude distributions (6-8m)
v Inh neuron amplitude distributions (6-8m)
Events (%)
50
25
0
1.25
1.75
2.25
2.75
3.25
3.75
4.25
4.75
Amplitude (ΔF/F0)
w Amplitude per mouse vs plaque load (3-8m)
EXC
INH
<0.5
0.5-1.0
>1.5
10^-3 plaques / μm^2
1.4
1.0
0.6
Amplitude (Norm to WT)
x Amplitude vs distance to plaque (3-8m)
0-10
10-20
20-30
>30
Distance to plaque (μm)
0.8
0.0
-0.8
Diff with WT (z-score)
y Motor Cortex (4-6m)
WT
EXC App^NL-G-F
INH App^NL-G-F
Normalised
1.5
1.0
0.5
AUC
Freq
Amp
z Methoxy-X04
MO 4-6m
V1 4-6m
aa Exc-Exc network size (MO)
App^NL-G-F (4-6m)
WT (4-6m)
Fraction of associated neurons
ab Inh-Exc network size (MO)

Supplementary Fig. 1 | Dysregulated resting-state activity at excitatory and inhibitory neurons in a mouse model of amyloidosis.

a-h, Average frequency (**a-d**) and amplitude (**e-h**) per mouse measured at 3-4m (left) and 6-8m (right) in WT (black) and $App^{NL\text{-}G\text{-}F}$ (blue) *Thy-1-GCaMP6s* mice from broad parcellated regions in Figure 1c. **i**, Density of methoxy-X04-positive plaques in 3-4m (left) and 6-8m (right) $App^{NL\text{-}G\text{-}F}$ mice. **j**, Cortical region showing immunofluorescence labelling of c-Fos (green), methoxy-X04 (magenta), and MOAB-2 (cyan) in $App^{NL\text{-}G\text{-}F}$ mice. Scale bar: 20 µm. **k-m**, Normalised (to V1) fluorescence intensity of c-Fos (**k**), methoxy-X04 (**l**), and MOAB-2 (**m**) in the visual (black) and motor (orange) cortices of 6-8m $App^{NL\text{-}G\text{-}F}$ mice. **n**, Area under the curve (AUC) of the $\Delta F/F_0$ trace of excitatory (left) and inhibitory (right) cells per mouse in 3-8m in WT (black) and $App^{NL\text{-}G\text{-}F}$ (blue, red) mice. **o-p**, Average amplitude of calcium events measured in excitatory (**o**, blue) and inhibitory (**p**, red) neurons in $App^{NL\text{-}G\text{-}F}$ mice at 3-6m (left) and 6-8m (right) and age-matched WT (black) animals. **q**, Amplitude of calcium events at excitatory (left) and inhibitory (right) cells per mouse in 3-8m in WT (black) and $App^{NL\text{-}G\text{-}F}$ (blue, red) mice. **r-s**, Frequency of calcium events measured in excitatory (**r**, blue) and inhibitory (**s**, red) neurons in $App^{NL\text{-}G\text{-}F}$ mice at 3-6m (left) and 6-8m (right) and age-matched WT (black) animals. **t**, Frequency of calcium events at excitatory (left) and inhibitory (right) cells per mouse in 3-8m in WT (black) and $App^{NL\text{-}G\text{-}F}$ (blue, red) mice. **u-v**, Percentage of calcium events of increasing amplitude in excitatory (**u**) and inhibitory (**v**) neurons in 6-8m in WT (black) and $App^{NL\text{-}G\text{-}F}$ (blue, red) mice. **w**, Heatmap showing normalised (to WT) excitatory (top) and inhibitory (bottom) neuron amplitude in 3-8m $App^{NL\text{-}G\text{-}F}$ mice with increasing plaque load. **x**, Heatmaps showing difference ($App^{NL\text{-}G\text{-}F}$-WT) in the z-scored (to WT) amplitude values of excitatory (top) and inhibitory (bottom) neurons located at increasing distances from methoxy-X04-labelled plaques in $App^{NL\text{-}G\text{-}F}$ mice. **y**, Normalised (to WT) area under the curve (AUC), mean frequency (Freq), and amplitude (Amp) of calcium events at excitatory (left) and inhibitory (right) cells

per mouse in 4-6m WT (black) and *App*$^{NL\text{-}G\text{-}F}$ (blue, red) mice. **z**, Density of methoxy-X04-positive plaques in the motor (left) and visual (right) cortex of 4-6m *App*$^{NL\text{-}G\text{-}F}$ mice. **aa-ab**, Fraction of functionally associated neurons in 4-6m *App*$^{NL\text{-}G\text{-}F}$ (blue, red) and WT (black) mice for assemblies comprising excitatory neurons alone (**aa**, blue), and inhibitory and excitatory neurons (**ab**, red). The data in figure panels a-h, i and n-x, j-m, and y-ab was obtained from 26, 19, 3 and 8 animals, respectively. Error bars: median (dotted: mean) ± IQR ± 5th-95th percentile (a-h, o-p, r-s), mean ± SEM (i,n,q,t-z). *p<0.05, *p<0.01, ***p<0.001; NS, not significant. Detailed statistics are reported in Supp. Data S7. Source data are provided as a Source Data file.

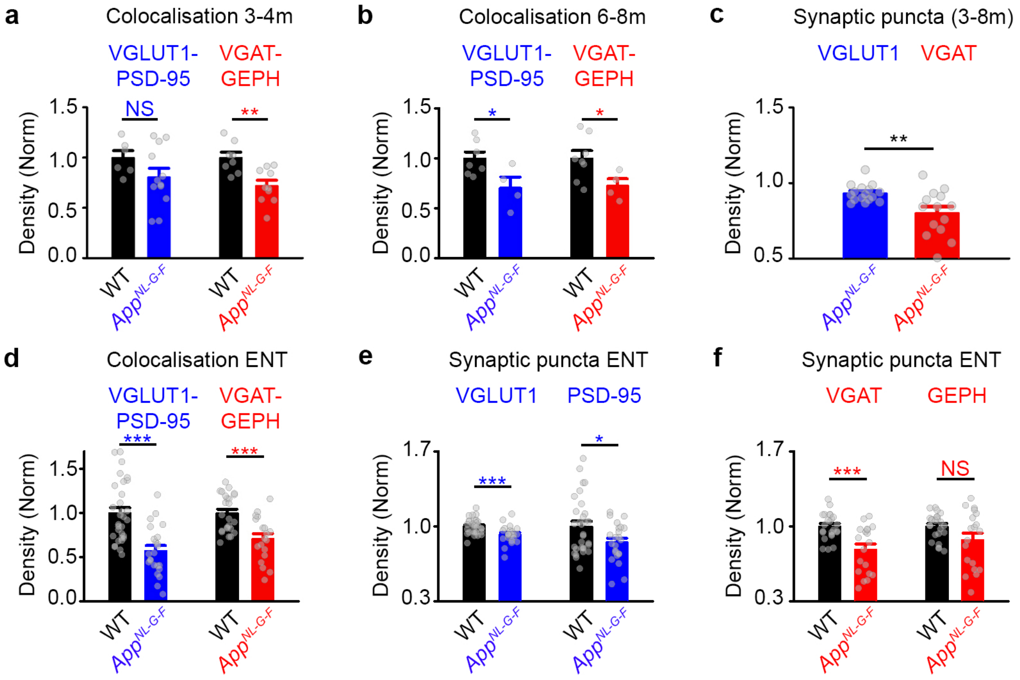
a
Colocalisation 3-4m
VGLUT1-PSD-95
VGAT-GEPH
NS
**
Density (Norm)
1.5
1.0
0.5
0.0
WT
App^NL-G-F
b
Colocalisation 6-8m
VGLUT1-PSD-95
VGAT-GEPH
*
c
Synaptic puncta (3-8m)
VGLUT1
VGAT
**
d
Colocalisation ENT
***
e
Synaptic puncta ENT
VGLUT1
PSD-95
1.7
0.3
f
Synaptic puncta ENT
VGAT
GEPH

Supplementary Fig. 2 | Presynaptic GABAergic vulnerability in *App^NL-G-F^* mouse cortex.

a-b, Normalised (to WT) colocalised excitatory (left) and inhibitory (right) synaptic puncta in $App^{NL\text{-}G\text{-}F}$ (blue, red) and age-matched WT (black) mice at 3-4m (**a**) and 6-8m (**b**). **c**, Normalised (to WT) density of VGLUT1 (blue) and VGAT (red) in 3-8m $App^{NL\text{-}G\text{-}F}$ mice. **d**, Normalised (to WT) colocalised glutamatergic (left) and GABAergic (right) synaptic puncta in 3-8m $App^{NL\text{-}G\text{-}}F$ (blue, red) and age-matched WT (black) mice in the entorhinal cortex (ENT). **e-f**, Normalised (to WT) density of excitatory (**e**) and inhibitory (**f**) pre- (left) and post- (right) synaptic proteins in 3-8m $App^{NL\text{-}G\text{-}}F$ (blue, red) and age-matched WT mice (black) in the entorhinal cortex (ENT). The data in this figure was obtained from 17 animals. Error bars: mean ± SEM, *p<0.05, **p<0.01, ***p<0.001; NS, not significant. Detailed statistics are reported in Supp. Data S8. Source data are provided as a Source Data file.

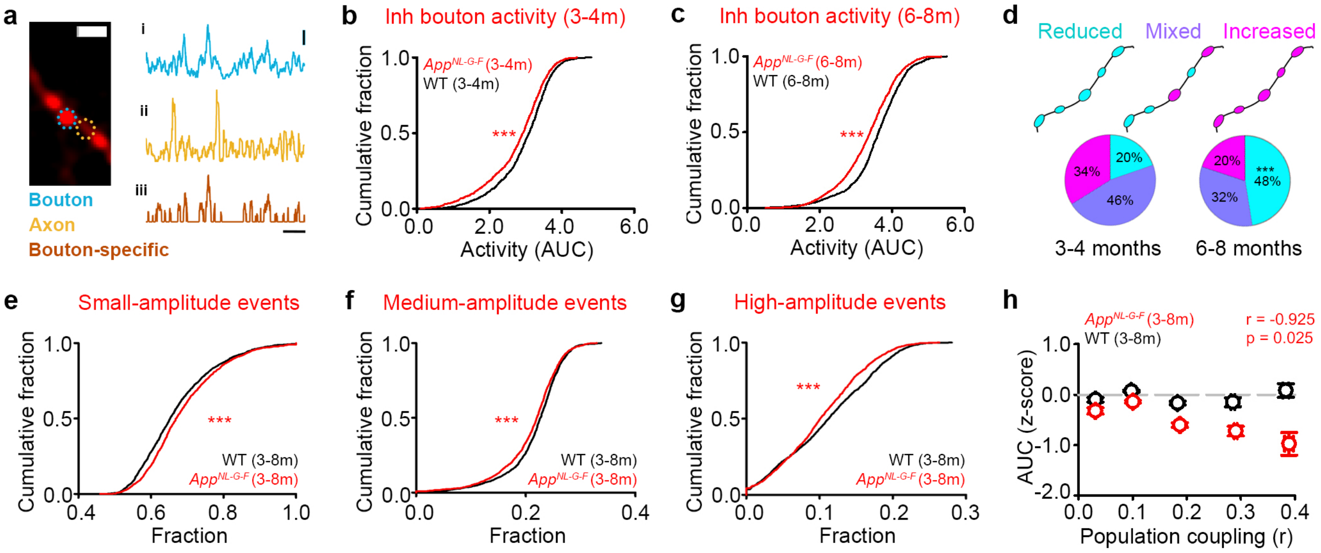

a
Bouton
Axon
Bouton-specific
i
ii
iii
b
Inh bouton activity (3-4m)
AppNL-G-F (3-4m)
WT (3-4m)
***
Cumulative fraction
Activity (AUC)
c
Inh bouton activity (6-8m)
AppNL-G-F (6-8m)
WT (6-8m)
***
Cumulative fraction
Activity (AUC)
d
Reduced
Mixed
Increased
34%
20%
46%
20%
***
48%
32%
3-4 months
6-8 months
e
Small-amplitude events
***
WT (3-8m)
AppNL-G-F (3-8m)
Cumulative fraction
Fraction
f
Medium-amplitude events
***
WT (3-8m)
AppNL-G-F (3-8m)
Cumulative fraction
Fraction
g
High-amplitude events
***
WT (3-8m)
AppNL-G-F (3-8m)
Cumulative fraction
Fraction
h
AppNL-G-F (3-8m)
WT (3-8m)
r = -0.925
p = 0.025
AUC (z-score)
Population coupling (r)

Supplementary Fig. 3 | Functional signatures of dysregulated GABAergic bouton activity in *App*$^{NL\text{-}G\text{-}F}$ mice.

a, Extraction of $\Delta F/F_0$ calcium signal from axonal bouton. Same-size regions of interest are placed around axonal bouton (blue) and adjacent axon (yellow). Scale bar: 2 µm. Normalised bouton (i) and axon (ii) signals are subtracted from each other, and a threshold is applied to extract the bouton-specific signal (iii). Scale bars: 0.5 $\Delta F/F_0$ and 5 s. **b-c**, Calcium-mediated activity (AUC) at GABAergic boutons during resting-state at 3-4m (**b**) and 6-8m (**c**) in WT (black) and *App*$^{NL\text{-}G\text{-}F}$ (red) mice. **d**, Percentage of axons where the activity of all measured GABAergic boutons was reduced (cyan), mixed (purple), or increased (magenta) relative to WT mean at 3-4m (left) and 6-8m (right) in *App*$^{NL\text{-}G\text{-}F}$. **e-g**, Fraction of small- (<0.45) (**e**), medium- (0.45-0.65) (**f**), and high- (>0.65) (**g**) amplitude calcium events in low-activity (AUC z-score<0) GABAergic boutons in 3-8m *App*$^{NL\text{-}G\text{-}F}$ (red) and WT (black) mice. **h**, Resting-state activity (AUC) of GABAergic boutons with increasing levels of population coupling in 3-8m *App*$^{NL\text{-}G\text{-}F}$ (red) and age-matched WT (black) mice. The data in this figure was obtained from 27 animals. Error bars: mean ± SEM. ***p<0.001. Detailed statistics are reported in Supp. Data S9. Source data are provided as a Source Data file.

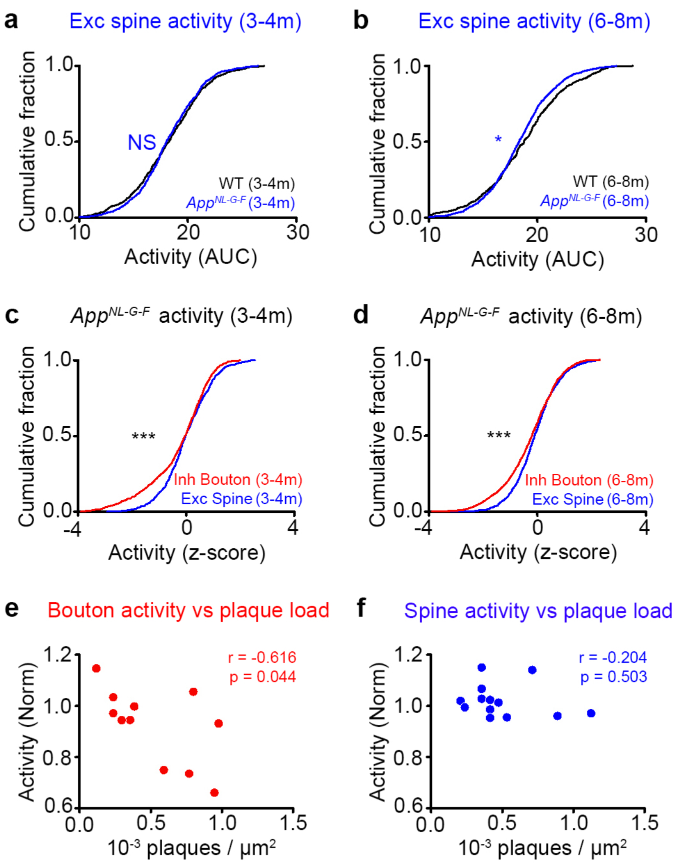

a
Exc spine activity (3-4m)
Cumulative fraction
NS
WT (3-4m)
App^NL-G-F (3-4m)
Activity (AUC)
b
Exc spine activity (6-8m)
Cumulative fraction
*
WT (6-8m)
App^NL-G-F (6-8m)
Activity (AUC)
c
App^NL-G-F activity (3-4m)
Cumulative fraction
***
Inh Bouton (3-4m)
Exc Spine (3-4m)
Activity (z-score)
d
App^NL-G-F activity (6-8m)
Cumulative fraction
***
Inh Bouton (6-8m)
Exc Spine (6-8m)
Activity (z-score)
e
Bouton activity vs plaque load
Activity (Norm)
r = -0.616
p = 0.044
10^-3 plaques / μm^2
f
Spine activity vs plaque load
Activity (Norm)
r = -0.204
p = 0.503
10^-3 plaques / μm^2

Supplementary Fig. 4 | Functional signatures of dysregulated synaptic activity in *App*$^{NL\text{-}G\text{-}F}$ mice.

a-b, Calcium activity (AUC) of dendritic spines at 3-4m (**a**) and 6-8m (**b**) in WT (black) and *App*$^{NL\text{-}G\text{-}F}$ (blue) mice. **c-d**, Z-scored (to WT) calcium activity (AUC) in GABAergic boutons (red) or excitatory dendritic spines (blue) during resting-state activity at 3-4m (**c**) and 6-8m (**d**) in *App*$^{NL\text{-}G\text{-}F}$ mice. **e-f**, Correlation between GABAergic bouton (**e**) and excitatory dendritic spine (**f**) activity (norm to WT) and median plaque density per mouse in 3-8m *App*$^{NL\text{-}G\text{-}F}$ mice. The data in this figure was obtained from 32 animals. *$p<0.05$, ***$p<0.001$; NS, not significant. Detailed statistics are reported in Supp. Data S10. Source data are provided as a Source Data file.

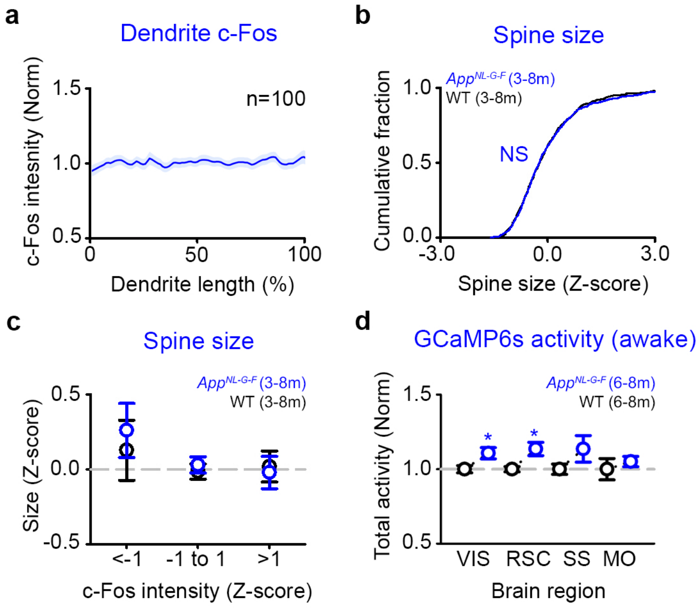

a
Dendrite c-Fos
c-Fos intesity (Norm)
1.5
1.0
0.5
n=100
0
50
100
Dendrite length (%)
b
Spine size
Cumulative fraction
App^NL-G-F (3-8m)
WT (3-8m)
1.0
0.5
0.0
NS
-3.0
0.0
3.0
Spine size (Z-score)
c
Spine size
App^NL-G-F (3-8m)
WT (3-8m)
Size (Z-score)
0.5
0.0
-0.5
<-1
-1 to 1
>1
c-Fos intensity (Z-score)
d
GCaMP6s activity (awake)
App^NL-G-F (6-8m)
WT (6-8m)
Total activity (Norm)
1.5
1.0
0.5
*
*
VIS
RSC
SS
MO
Brain region

Figure S5

Supplementary Fig. 5 | *App*$^{NL\text{-}G\text{-}F}$ mice show increased activity in posterior cortical regions.

a, c-Fos fluorescence normalised to dendrite mean, along relative dendrite length. **b**, Z-scored (to WT) dendritic spine size from 3-8m *App*$^{NL\text{-}G\text{-}F}$ mice (blue) and age-matched WT (black). **c**, Z-scored (to WT) spine size at dendrites with increasing levels of c-Fos expression in 3-8m *App*$^{NL\text{-}G\text{-}F}$ (blue) and age-matched WT (black) mice. **d**, Normalised calcium-mediated activity (freq x amp) per mouse in visual (VIS), retrosplenial (RSC), somatosensory (SS) and motor (MO) areas at 6-8m in Thy-1-GCaMP6s (black) and *App*$^{NL\text{-}G\text{-}F}$ x Thy-1-GCaMP6s (blue) awake mice. The data in figure panels a-c, and d was obtained from 20 and 13 animals, respectively. Error bars: mean ± SEM. *$p<0.05$; NS, not significant. Detailed statistics are reported in Supp. Data S11. Source data are provided as a Source Data file.

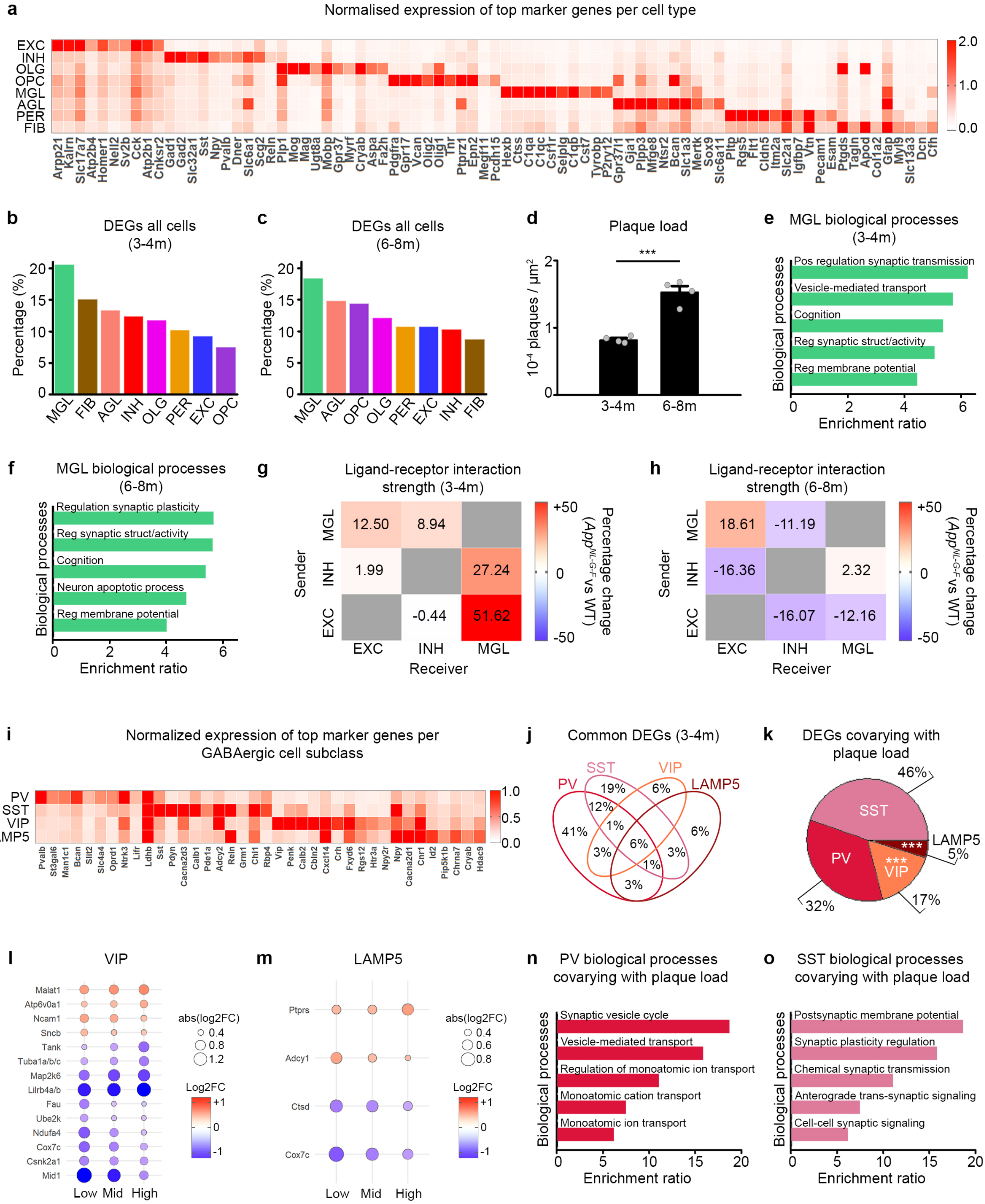

a
Normalised expression of top marker genes per cell type
EXC
INH
OLG
OPC
MGL
AGL
PER
FIB
2.0
1.0
0.0
Arpp21 Kalrn Slc17a7 Atp2b4 Homer1 Nell2 Sv2b Cck Atp2b1 Cnksr2 Gad1 Gad2 Slc32a1 Sst Npy Pvalb Dner Slc6a1 Scg2 Reln Plp1 Mog Mag Ugt8a Mobp Gpr37 Myrf Cryab Aspa Fa2h Pdgfra Gpr17 Vcan Olig2 Olig1 Tnr Ptprz1 Epn2 Megf11 Pcdh15 Hexb Ctss C1qa C1qc Csf1r Selplg C1qb Cst7 Tyrobp P2ry12 Gpr37l1 Gja1 Plpp3 Mfge8 Ntsr2 Bcan Slc1a3 Mertk Sox9 Slc6a11 Pltp Rgs5 Flt1 Cldn5 Itm2a Slc2a1 Igfbp7 Vtn Pecam1 Esam Ptgds Tagln Apod Col1a2 Gfap Myl9 Slc13a3 Dcn Cfh
b
DEGs all cells (3-4m)
Percentage (%)
MGL FIB AGL INH OLG PER EXC OPC
c
DEGs all cells (6-8m)
Percentage (%)
MGL AGL OPC OLG PER EXC INH FIB
d
Plaque load
10⁻⁴ plaques / µm²
***
3-4m
6-8m
e
MGL biological processes (3-4m)
Biological processes
Pos regulation synaptic transmission
Vesicle-mediated transport
Cognition
Reg synaptic struct/activity
Reg membrane potential
Enrichment ratio
f
MGL biological processes (6-8m)
Biological processes
Regulation synaptic plasticity
Reg synaptic struct/activity
Cognition
Neuron apoptotic process
Reg membrane potential
Enrichment ratio
g
Ligand-receptor interaction strength (3-4m)
Sender
MGL 12.50 8.94
INH 1.99 27.24
EXC -0.44 51.62
EXC INH MGL
Receiver
+50
0
-50
Percentage change (App^NL-G-F vs WT)
h
Ligand-receptor interaction strength (6-8m)
Sender
MGL 18.61 -11.19
INH -16.36 2.32
EXC -16.07 -12.16
EXC INH MGL
Receiver
+50
0
-50
Percentage change (App^NL-G-F vs WT)
i
Normalized expression of top marker genes per GABAergic cell subclass
PV
SST
VIP
LAMP5
1.0
0.5
0.0
Pvalb St3gal6 Man1c1 Bcan Slit2 Slc4a4 Oprd1 Ntrk3 Lifr Ldhb Sst Pdyn Cacna2d3 Calb1 Pde1a Adcy2 Reln Grm1 Chl1 Rbp4 Vip Penk Calb2 Cbln2 Cxcl14 Crh Fxyd6 Rgs12 Htr3a Npy2r Npy Cacna2d1 Cnr1 Id2 Pip5k1b Chrna7 Cryab Hdac9
j
Common DEGs (3-4m)
PV SST VIP LAMP5
41% 19% 12% 6% 1% 6% 3% 6% 3% 1% 3%
k
DEGs covarying with plaque load
SST 46%
LAMP5 5% ***
VIP 17% ***
PV 32%
l
VIP
Malat1 Atp6v0a1 Ncam1 Sncb Tank Tuba1a/b/c Map2k6 Lilrb4a/b Fau Ube2k Ndufa4 Cox7c Csnk2a1 Mid1
Low Mid High
abs(log2FC) 0.4 0.8 1.2
Log2FC +1 0 -1
m
LAMP5
Ptprs Adcy1 Ctsd Cox7c
Low Mid High
abs(log2FC) 0.4 0.6 0.8
Log2FC +1 0 -1
n
PV biological processes covarying with plaque load
Biological processes
Synaptic vesicle cycle
Vesicle-mediated transport
Regulation of monoatomic ion transport
Monoatomic cation transport
Monoatomic ion transport
Enrichment ratio
o
SST biological processes covarying with plaque load
Biological processes
Postsynaptic membrane potential
Synaptic plasticity regulation
Chemical synaptic transmission
Anterograde trans-synaptic signaling
Cell-cell synaptic signaling
Enrichment ratio

Supplementary Fig. 6 | Transcriptomic changes during amyloidosis in the *App*$^{NL\text{-}G\text{-}F}$ mouse.

a, Normalised expression of top 10 marker genes per cell type. **b-c**, Percentage of DEGs for the main cell types when comparing *App*$^{NL\text{-}G\text{-}F}$ and WT at 3-4m (**b**) and 6-8m (**c**). Excitatory (EXC) and inhibitory (INH) neurons, oligodendroglial cells (OLG), oligodendrocyte precursor cells (OPC), microglia (MGL), astroglia (AGL), pericytes (PER), and fibroblasts (FIB). **d**, Plaque density per animal in *App*$^{NL\text{-}G\text{-}F}$ mice at 3-4m (left) and 6-8m (right). **e-f,** Biological processes associated with DEGs in MGL at 3-4m (**e**) and 6-8m (**f**). **g-h,** Percentage change in inferred ligand-receptor interaction strength (estimated based on probability weight) between MGL, INH, and EXC cells in *App*$^{NL\text{-}G\text{-}F}$ vs WT mice (red denotes increased and blue denotes decreased in *App*$^{NL\text{-}G\text{-}F}$) at 3-4m (**g**) and 6-8m (**h**). **i**, Normalised expression of top 10 marker genes per GABAergic cell subclass. **j,** Percentage of DEGs shared across different GABAergic subclasses. Empty overlap denotes 0%. **k**, Percentage of DEGs that covary with plaque load when comparing *App*$^{NL\text{-}G\text{-}F}$ and WT across GABAergic subclasses. Comparisons against SST shown for each subclass. **l-m**, DEGs that covary with plaque load for VIP (**l**) and LAMP5 (**m**) neurons. **n-o**, Biological processes associated with DEGs that covary with plaque load in PV (**n**) and SST (**o**) neurons. All panels DEGs: MAST, adj. $p < 0.05$ and abs(log2FC) > 0.25. Biological processes (**e-f**, **n-o**): ORA; BH-FDR < 0.05. The data in this figure was obtained from 16 animals. Error bars: mean ± SEM. ***p<0.001. Detailed statistics are reported in Supp. Data S12. Source data are provided as a Source Data file.
