# Supplementary material for: Selective weakening of population-coupled synaptic activity in vivo in a mouse model of amyloid-beta pathology
Source: Nat Commun. 2026 Mar 7;17:3646. doi: 10.1038/s41467-026-69866-3 (PMC13096637; doi:10.1038/s41467-026-69866-3)
Supplement: Supplementary file 2 — Description of Additional Supplementary Files [file 41467_2026_69866_MOESM2_ESM.pdf]

**Description of Additional Supplementary Files**

Supplementary Data 1: Contains detailed statistical comparisons for Figure 1.

Supplementary Data 2: Contains detailed statistical comparisons for Figure 2.

Supplementary Data 3: Contains detailed statistical comparisons for Figure 3.

Supplementary Data 4: Contains detailed statistical comparisons for Figure 4.

Supplementary Data 5: Contains detailed statistical comparisons for Figure 5.

Supplementary Data 6: Contains detailed statistical comparisons for Figure 6.

Supplementary Data 7: Contains detailed statistical comparisons for Supp. Figure 1.

Supplementary Data 8: Contains detailed statistical comparisons for Supp. Figure 2.

Supplementary Data 9: Contains detailed statistical comparisons for Supp. Figure 3.

Supplementary Data 10: Contains detailed statistical comparisons for Supp. Figure 4.

Supplementary Data 11: Contains detailed statistical comparisons for Supp. Figure 5.

Supplementary Data 12: Contains detailed statistical comparisons for Supp. Figure 6.

Supplementary Data 13: Contains the list of chemicals and reagents used for immunolabelling.

Supplementary Data 14: Contains detailed experimental conditions for immunofluorescence assays.
