# Supplementary material for: Selective weakening of population-coupled synaptic activity in vivo in a mouse model of amyloid-beta pathology
Source: Nat Commun. 2026 Mar 7;17:3646. doi: 10.1038/s41467-026-69866-3 (PMC13096637; doi:10.1038/s41467-026-69866-3)
Supplement: Supplementary file 4 — Reporting Summary [file 41467_2026_69866_MOESM4_ESM.pdf]

nature portfolio

Corresponding author(s): Samuel Barnes

Last updated by author(s): Feb 10, 2026

Reporting Summary

Nature Portfolio wishes to improve the reproducibility of the work that we publish. This form provides structure for consistency and transparency in reporting. For further information on Nature Portfolio policies, see our Editorial Policies and the Editorial Policy Checklist.

Statistics

For all statistical analyses, confirm that the following items are present in the figure legend, table legend, main text, or Methods section.

n/a	Confirmed	
☐	☒	The exact sample size (n) for each experimental group/condition, given as a discrete number and unit of measurement
☐	☒	A statement on whether measurements were taken from distinct samples or whether the same sample was measured repeatedly
☐	☒	The statistical test(s) used AND whether they are one- or two-sided<br>*Only common tests should be described solely by name; describe more complex techniques in the Methods section.*
☐	☒	A description of all covariates tested
☐	☒	A description of any assumptions or corrections, such as tests of normality and adjustment for multiple comparisons
☐	☒	A full description of the statistical parameters including central tendency (e.g. means) or other basic estimates (e.g. regression coefficient) AND variation (e.g. standard deviation) or associated estimates of uncertainty (e.g. confidence intervals)
☐	☒	For null hypothesis testing, the test statistic (e.g. F, t, r) with confidence intervals, effect sizes, degrees of freedom and P value noted<br>*Give P values as exact values whenever suitable.*
☒	☐	For Bayesian analysis, information on the choice of priors and Markov chain Monte Carlo settings
☐	☒	For hierarchical and complex designs, identification of the appropriate level for tests and full reporting of outcomes
☐	☒	Estimates of effect sizes (e.g. Cohen's d, Pearson's r), indicating how they were calculated

Our web collection on statistics for biologists contains articles on many of the points above.

Software and code

Policy information about availability of computer code

Data collection	Statistical analyses were performed in MATLAB (version R2021b), SigmaPlot (version 14.0, Systat Software), ImageJ (Fiji, version 1.53t), or R (version 4.3.1).
Data analysis	Statistical analyses were performed in MATLAB (version R2021b), or SigmaPlot (version 14.0, Systat Software), ImageJ (Fiji, version 1.53t), or R (version 4.3.1).

For manuscripts utilizing custom algorithms or software that are central to the research but not yet described in published literature, software must be made available to editors and reviewers. We strongly encourage code deposition in a community repository (e.g. GitHub). See the Nature Portfolio guidelines for submitting code & software for further information.

Data

Policy information about availability of data

All manuscripts must include a data availability statement. This statement should provide the following information, where applicable:

- Accession codes, unique identifiers, or web links for publicly available datasets
- A description of any restrictions on data availability
- For clinical datasets or third party data, please ensure that the statement adheres to our policy

Source data are provided with this paper. Data associated with this paper has been deposited at 10.5281/zenodo.18369811. Due to large file size, other datasets generated in this study are also available from the author upon request.

Human research participants

Policy information about studies involving human research participants and Sex and Gender in Research.

Reporting on sex and gender	n/a
Population characteristics	n/a
Recruitment	n/a
Ethics oversight	n/a

Note that full information on the approval of the study protocol must also be provided in the manuscript.

Field-specific reporting

Please select the one below that is the best fit for your research. If you are not sure, read the appropriate sections before making your selection.

[x] Life sciences [ ] Behavioural & social sciences [ ] Ecological, evolutionary & environmental sciences

For a reference copy of the document with all sections, see nature.com/documents/nr-reporting-summary-flat.pdf

Life sciences study design

All studies must disclose on these points even when the disclosure is negative.

Sample size	Sample size was based on pilot data and is reported in statistical tables.
Data exclusions	In vivo imaging data were excluded if motion artifacts could not be corrected or if cells exhibited filled nuclei due to GCaMP overexpression.
Replication	Findings were replicated using both technical and biological replicates. Furthermore major biological results were reproduced using additional techniques, such as changes in synaptic density being measured both ex vivo with immunofluorescence and in vivo with synaptic structural imaging.
Randomization	Data was randomized among investigators for analysis.
Blinding	All analysis was conducted blind to the condition of the acquired data files.

Reporting for specific materials, systems and methods

We require information from authors about some types of materials, experimental systems and methods used in many studies. Here, indicate whether each material, system or method listed is relevant to your study. If you are not sure if a list item applies to your research, read the appropriate section before selecting a response.

Materials & experimental systems

n/a	Involved in the study	
[ ]	[x]	Antibodies
[x]	[ ]	Eukaryotic cell lines
[x]	[ ]	Palaeontology and archaeology
[ ]	[x]	Animals and other organisms
[x]	[ ]	Clinical data
[x]	[ ]	Dual use research of concern

Methods

n/a	Involved in the study	
[x]	[ ]	ChIP-seq
[x]	[ ]	Flow cytometry
[x]	[ ]	MRI-based neuroimaging

Antibodies

Antibodies used	Details about antibodies and their concentrations can be found in Supplementary Tables 13-14.
Validation	Quality control information and relevant citations are available at manufacturer's website. For anti-VGLUT1: https://sysy.com, http://file.yizimg.com/313862/2019032121221593.pdf; for anti-PSD-95: https://www.antibodiesinc.com/products/anti-psd-95-antibody-k28-43-75-028; for anti-VGAT: https://sysy.com/product/131011; for anti-gephyrin: https://sysy.com, https://www.citeab.com/antibodies/509387-147-002-gephyrin; for anti-c-Fos: https://www.abcam.com/en-us/products/primary-antibodies/c-fos-antibody-bsa-free-ab190289?srsltid=AfmBOoqQ2dJQNfeRExROL5ozh8pgnkEsmLtoabnUASulTauytgPSzDeV; for MOAB-2: https://www.novusbio.com/products/beta-amyloid-antibody-moab-2_nbp2-13075?

srsltid=AfmBOooAMxW30OGKX2Qq7PHpmpRq30f8eaOnds6tb2WIBKBsIz_pLo6h; for anti-PV: https://sysy.com/product/195006; for anti-SST: https://www.sigmaaldrich.com/GB/en/product/mm/mab354?srsltid=AfmBOoq6HGC5KkorMYMFGy_lxa_53W_o_WMKx8r9-h6milz1S4jCQsKp

Animals and other research organisms

Policy information about studies involving animals; ARRIVE guidelines recommended for reporting animal research, and Sex and Gender in Research

Laboratory animals	We used adult (P60-240) male and female mice on a C57BL/J6 background. For most experiments, we used homozygous AppNL-G-F mice (Saito et al., 2014. Nat Neurosci), and age-matched C57BL/J6 mice served as controls. For mesoscopic imaging, AppNL-G-F mice were crossed with Thy-1-GCaMP6s98 mice (JAX stock #007788, The Jackson Laboratory, Bar Harbor, ME) and Thy-1-GCaMP6s line served as controls.
Wild animals	We did not use wild animals.
Reporting on sex	All mice were sex and age matched between experimental groups.
Field-collected samples	Did not involve samples from the field.
Ethics oversight	Experiments were conducted according to the United Kingdom Animals (Scientific Procedures) Act 1986.

Note that full information on the approval of the study protocol must also be provided in the manuscript.
